# Non-insulin-based insulin resistance indices predict early neurological deterioration in elderly and middle-aged acute ischemic stroke patients in Northeast China

**DOI:** 10.1038/s41598-024-66881-6

**Published:** 2024-07-12

**Authors:** Jia Wang, Hao Tang, Jianan Tian, Yibo Xie, Yun Wu

**Affiliations:** Department of Neurology, The Second Affiliated Hospital of Harbin Medical University, No.148. Baojian Road, NanGangDistrict, Harbin City, Heilongjiang Province China

**Keywords:** Triglyceride glucose-body mass index, insulin resistance, early neurological deterioration, Acute ischemic stroke, Diseases of the nervous system, Endocrinology, Neurology

## Abstract

Insulin resistance (IR) has a strong association with acute ischemic stroke (AIS) occurrence and poor prognosis of afflicted patients. However, the relation between early neurological deterioration (END) risk and IR in elderly and middle-aged patients remains to be thoroughly studied. Here, we investigated the relationship between four indicators of IR and the risk of END in middle-aged patients patients with AIS. The study retrospectively analyzed 1696 elderly and middle-aged patients having AIS between January 2019 and June 2023. Within 7 days of admission, the patients were then stratified relying upon alternations in the National Institutes of Health Stroke Scale. Subsequently, we employed logistic regression analyses for assessing each index correlation with END on the basis of the tertiles of TyG index (TyGI), triglyceride to high-density lipoprotein ratio (TG/HDL), TyG-BMI, alongside IR metabolic score (METS-IR). These four indicators were significantly heightened in the END group (n = 680) in comparison to the non-END group (n = 1016). When grouping using tertiles, the four aforementioned indicators emerged as independent risk factors for END occurrence, whether or not adjusted for confounding factors. The results revealed a progressive elevation in END occurrence risk with the rise in the tertile of each indicator. Finally, we utilized receiver operating characteristic (ROC) curves for assessing the indicators' predictive power. TyG-BMI, TyGI, TG/HDL, and METS-IRs’ area under the curve (AUC) were, respectively, 0.736 (95% CI: 0.712–0.761; *P* < 0.001), 0. 694 (95% CI: 0.668–0.721; *P* < 0.001), 0.684 (95% CI: 0.658–0.711; *P* < 0.001), and 0.722 (95% CI: 0.697–0.747; *P* < 0.001). IR is associated with END risk in middle-aged AIS patients. TyG-BMI, TyGI, TG/HDL, and METS-IR are independent risk factors of END in elderly and middle-aged AIS patients. Simultaneously, these four IR indicators have significant predictive power for END.

## Introduction

Stroke places an enormous burden on healthcare systems worldwide, as it is the primary reason for morbidity and mortality in the majority of countries^1,2^. Because of the acute onset and narrow treatment window of stroke, a significant proportion of patients experience early neurological deterioration (END), which affects long-term patient outcomes^3,4^, or worse, functional deterioration with age^5^, even when patients seek timely medical attention in the hyperacute or acute phase. As the aging population continues to grow, the burden will escalate over time. Consequently, early detection and treatment of END is essential in elderly and middle-aged patients suffering from acute ischemic stroke (AIS).

Insulin resistance (IR) constitutes an impaired physiological response of the target tissue to insulin stimulation^6^. Besides being the basis for type II diabetes pathogenesis, IR is the prevalent pathophysiology for various metabolic diseases that include ischemic stroke^7^, coronary heart disorder (CHD)^8,9^, and hyperuricemia^10,11^. Being the gold standard for assessing IR, the high insulin-normal glucose clamp represents both an intricate and expensive method^12^, which limits its use in clinical practice^13^. Accordingly, researching simple and efficient proxies is essential. Earlier studies have shown that biochemical test-derived measures, including IR metabolic score (METS-IR), triglyceride-glucose index (TyGI), along triglyceride to high-density lipoprotein ratio (TG/HDL), can serve as IR level markers^14–18^. Combining body mass index (BMI) with TyG has succeeded in improving IR prediction^19^. In a national prospective cohort study, significant alternations in TyG-BMI were independently correlated with stroke risk in older and middle-aged individuals^20^. However, it is uncertain whether the aforementioned non-insulin-dependent indicators of IR are associated with END development. Accordingly, we investigated the relationship between the aforementioned measures and END using a large clinical cohort of elderly AIS patients.

## Methods

### Study design and participants

**Figure 1:**
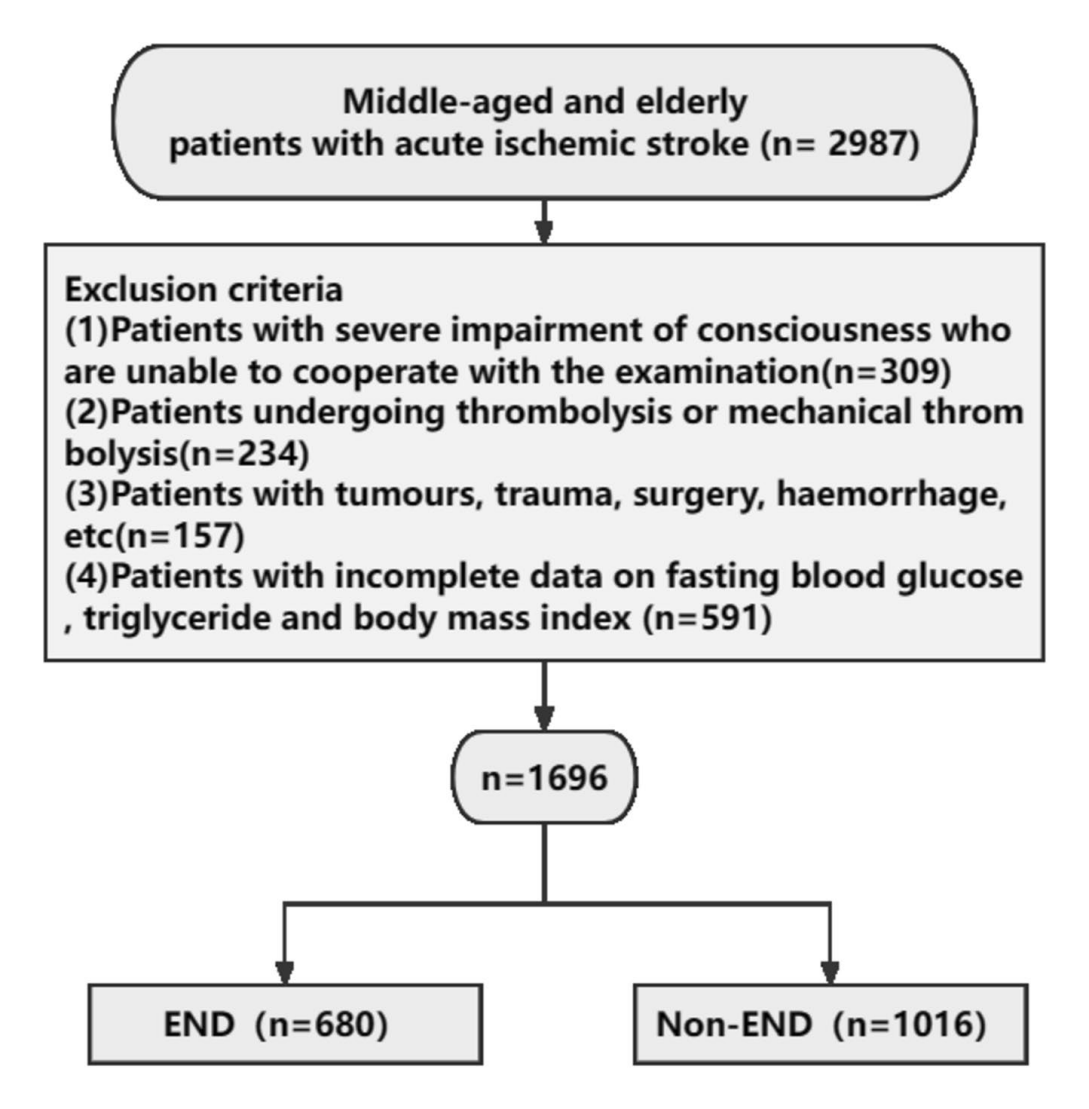
Study flowchart.

This retrospective study analyzed 1696 AIS patients admitted to the Second Hospital of Harbin Medical University between January 2019 and June 2023, with the human ethics committee of the same institution approving this study (NO. KY2023-162). Inclusion criteria: participants must be at least 45 years of age and have a magnetic resonance imaging diagnosis of AIS. Exclusion criteria: patients were severely impaired in consciousness, unable to cooperate with the study, had undergone thrombolysis or mechanical retrieval of a clot, had a tumor, trauma, surgery, hemorrhage, or incomplete data regarding BMI, fasting blood glucose (FBG), and triglyceride (TG). Figure 1 depicts the patient recruitment.

### Data collection

Our study obtained clinical data on the admission of AIS patients to this center: sex, age, weight, height, smoking and alcohol status, as well as diabetes mellitus (DM), hypertension (HTN), and CHD history. On the morning of the second day after enrolment, a healthcare professional drew venous blood from the patients in the fasting state. The following parameters for several indicators were immediately recorded: fasting glucose (FPG), c-reactive protein (CRP), homocysteine (HCY), total cholesterol (TC), TGs, low-density lipoprotein (LDL), and HDL. Furthermore, a designated and professionally trained physician daily evaluated the National Institutes of Health Stroke Scale (NIHSS) score to determine END presence or absence.

### Definitions

END: NIHSS score ≥ 2 through a week of enrollment in comparison to on enrollment^21^.

The formulae used to define various indicators are given below^16,18,22,23^:

$${\text{BMI }} = {\text{ weight}}\left( {{\text{kg}}} \right)/{\text{height}}^{{2}} \left( {{\text{m}}^{{2}} } \right)$$

$${\text{TyG}} = {\text{Ln }}\left[ {{\text{TG }}\left( {{\text{mg}}/{\text{dL}}} \right) \times {\text{FPG }}\left( {{\text{mg}}/{\text{dL}}} \right) \div {2}} \right]$$

$${\text{TyG}} - {\text{BMI}} = {\text{TyG}} \times {\text{BMI }}\left( {{\text{kg}}/{\text{m}}^{{2}} } \right)$$

$${\text{TG}}/{\text{HDL}} = {\text{TG }}\left( {{\text{mg}}/{\text{dl}}} \right) \div {\text{HDL }}\left( {{\text{mg}}/{\text{dl}}} \right)$$

$${\text{METS}} - {\text{IR}} = {\text{Ln }}\left[ {\left( {{2} \times {\text{FPG }}\left( {{\text{mg}}/{\text{dl}}} \right)} \right) + {\text{TG }}\left( {{\text{mg}}/{\text{dl}}} \right)} \right] \times {\text{BMI }}\left( {{\text{kg}}/{\text{m2}}} \right) \div {\text{Ln }}\left[ {{\text{HDL }}\left( {{\text{mg}}/{\text{dl}}} \right)} \right]$$

### Participant stratification

The patients were categorized into groups relying upon tertiles outlined below:

T1/T2/T3 groups: TyGI < 8.804, ≥ 8.804 to < 9.486, and ≥ 9.486, respectively.

B1/B2/B3 groups: TyG-BMI index < 219.881, ≥ 219.881 to < 262.937, and ≥ 262.937, respectively.

G1/G2/G3 groups: TG/HDL < 3.169, ≥ 3.169 to < 5.104, and ≥ 5.104, respectively.

M1/M2/M3 groups: METS-IR < 35.021, ≥ 35.021 to < 42.704, and ≥ 42.704, respectively.

### Statistical analysis

Statistical analysis was conducted through SPSS 26.0 (IBM Corp, New York, NY, USA). Regarding continuous variables, normality was first evaluated by utilizing the Shapiro–Wilk test, expressing the results as mean ± standard deviation for normality and medians (P25–P75) for non-normality while reporting the categorical variable data as numbers and percentages. The χ^2^ test was employed for categorical variables while utilizing the t-test, Mann–Whitney U, and Kruskal–Wallis H tests for continuous variables. The calculation of odds ratios (OR) and 95% confidence intervals (CI) was performed through logistic regression analysis (LRA) to analyze each indicator correlation with END. Each indicator predictive capability of END was determined through receiver operating characteristics (ROC) and area under the curve (AUC). Meanwhile, MedCalc was used for comparison of the differences in AUC of the indicators. The statistical analyses were all two-sided, with *P* < 0.5 indicating a significant difference.

### Ethics approval and consent to participate

The study was conducted in accordance with the Declaration of Helsinki and approved by the Second Affiliated Hospital of Harbin Medical University Ethical Review Committee (NO. KY2023-162). Informed consent was obtained from all patients.

## Results

### Baseline characteristics

Of the 1696 enrolled patients, 680 and 1016 were, respectively, allocated to END and non-END groups. END group patients possessed advanced age and a greater incidence of smoking, DM, and HTN (*P* < 0.05) in comparison to the non-END group. Furthermore, baseline TyGI, BMI, NIHSS, TyG-BMI, FPG, TG, TG/HDL, HCY, CRP, and METS-IR exhibited significantly highest levels in the END group *(P* < 0.05). Nonetheless, HDL levels were significantly the lowest in END patients (*P* < 0.05). The other indicators displayed nonsignificant discrepancies between both groups (Table 1).

**Table 1:** Patient characteristics on the basis of early neurological deterioration (END).

Characteristics	ALL (n = 1696 )	END (n = 680)	Non-END (n = 1016)	*P*-value
Age (years)	61 (56, 66)	63 (57, 68)	61 (56, 65)	0.001
Male (n, %)	1115 (65.70%)	467 (68.70%)	648 (63.80%)	0.037
Body mass index (kg/m^2^)	26.46 (22.86, 29.75)	28.70 (25.08, 32.02)	25.31 (21.94, 27.89)	< 0.001
Hypertension (n, %)	1008 (59.50%)	453 (66.60%)	555 (54.70%)	< 0.001
Diabetes mellitus (n, %)	711 (41.90%)	340 (50.00%)	371 (36.50%)	< 0.001
Coronary heart disease (N, %)	176 (10.40%)	68 (10.00%)	108 (10.60%)	0.677
Smoking (n, %)	664 (39.20%)	301 (44.30%)	363 (35.70%)	< 0.001
Drinking (n, %)	561 (33.1%)	216 (31.80%)	345 (34.00%)	0.347
Initial National Institutes of Health Stroke Scale	3 (1, 5)	3 (2, 5)	3 (1, 4)	< 0.001
Total cholesterol (mmol/l)	4.60 (3.94, 5.38)	4.64 (3.97, 5.31)	4.58 (3.93, 5.41)	0.762
Triglycerides (mmol/l)	1.81 (1.37, 2.58)	2.29 (1.62, 3.22)	1.62 (1.28, 2.12)	< 0.001
Fasting plasma glucose (mmol/l)	6.14 (5.29, 8.41)	7.48 (5.74, 10.92)	5.78 (5.17, 6.93)	< 0.001
High-density lipoprotein (mmol/l)	1.07 (0.89, 1.25)	1.03 (0.83, 1.23)	1.08 (0.92, 1.28)	< 0.001
Low-density lipoprotein (mmol/l)	2.77 (2.24, 3.36)	2.83 (2.23, 3.39)	2.74 (2.25, 3.33)	0.348
C-reactive protein (mg/l)	2.50 (1.15, 5.45)	2.79 (1.31, 6.33)	2.35 (1.06, 4.87)	< 0.001
Homocysteine (umol/l)	12.52 (10.06, 16.37)	12.82 (10.36, 17.01)	12.37 (9.91, 16.08)	0.008
Triglyceride-glucose index	9.09 (8.66, 9.76)	9.52 (8.91, 10.24)	8.92 (8.57, 9.37)	< 0.001
Triglyceride glucose-body mass index	240.51 (206.81, 278.99)	269.27 (232.59, 314.02)	227.16 (197.58, 254.33)	< 0.001
Metabolic score for insulin resistance	38.82 (32.91, 44.97)	43.13 (36.92, 50.23)	36.42 (31.22, 41.61)	< 0.001
Triglyceride to high-density lipoprotein ratio	3.97 (2.83, 29.75)	5.03 (3.39, 8.16)	3.50 (2.59, 4.86)	< 0.001

### Association between END and risk factors

Univariate LRA was performed with END as the dependent variable to examine the relation between the indicators and END, revealing that the assessed factors, except for CHD, drinking status, TC, and LDL, exhibited a significant relation to END risk (*P* < 0.05) (Table 2).

**Table 2:** Early neurological deterioration (END) and risk factor associations.

Characteristics	END		
	OR (95% CI)	B	P-value
Age (years)	1.020 (1.007, 1.034)	0.020	0.003
Sex			
Female	Reference		
Male	1.245 (1.013, 1.531)	0.219	0.037
Hypertension			
No	Reference		
Yes	1.654 (1.352, 2.024)	0.503	< 0.001
Diabetes mellitus			
No	Reference		
Yes	1.739 (1.427, 2.118)	0.553	< 0.001
Coronary heart disease			
No	Reference		
Yes	0.934 (0.678, 1.287)	− 0.068	0.677
Smoking			
No	Reference		
Yes	1.429 (1.172, 1.742)	0.357	< 0.001
Drinking			
No	Reference		
Yes	0.905 (0.736, 1.114)	− 0.099	0.347
Body mass index	1.170 (1.143, 1.197)	0.157	< 0.001
Initial National Institutes of Health Stroke Scale	1.094 (1.054, 1.135)	0.089	< 0.001
Total cholesterol (mmol/l)	0.974 (0.893, 1.063)	− 0.026	0.557
Triglycerides (mmol/l)	1.704 (1.542, 1.882)	0.533	< 0.001
Fasting plasma glucose (mmol/l)	1.282 (1.233, 1.333)	0.249	< 0.001
High-density lipoprotein (mmol/l)	0.631 (0.521, 0.764)	− 0.461	< 0.001
Low-density lipoprotein (mmol/l)	1.036 (0.927, 1.158)	0.035	0.533
C-reactive protein (mg/l)	1.049 (1.022, 1.077)	0.048	< 0.001
Homocysteine (umol/l)	1.018 (1.005, 1.030)	0.017	0.004
Triglyceride-glucose index	2.455 (2.142, 2.814)	0.898	< 0.001
Triglyceride glucose-body mass index	1.019 (1.017, 1.022)	0.019	< 0.001
Metabolic score for insulin resistance	1.100 (1.085, 1.115)	0.095	< 0.001
Triglyceride to high-density lipoprotein ratio	1.181 (1.141, 1.223)	0.167	< 0.001

### TyGI and END association

Through a multivariate LRA with END as the dependent variable, we examine the inherent relationships and interdependencies between the variables. Next, from the continuous and categorical variables perspective, correlations between indicators and END were observed, controlling for various risk factors. For each indicator, three models were developed based on the following rules. Our study adjusted Models 1/2/3 for no risk factors, the two most common risk factors (age and sex), and univariate LRA risk factors, respectively. The TyGI was significantly related to END occurrence when it was a continuous variable (*P* < 0.05). When the indicators were then grouped based on tertiles and used as categorical variables, the risk of END was found to be elevated in both T2/T3 compared to T1 in all three models (Table 3) (*P* < 0.05).

**Table 3:** TyGI and END association.

Variables	END					
	OR (95% CI)^a^	*P*-value	OR (95% CI)^b^	*P*-value	OR (95% CI)^c^	*P*-value
TyGI	2.455 (2.142, 2.814)	< 0.001	3.545 (2.905, 4.326)	< 0.001	2.818 (2.289, 3.469)	< 0.001
T1	Reference					
T2	1.435 (1.107, 1.859)	0.006	2.12 (1.571, 2.861)	< 0.001	3.783 (2.682, 5.335)	< 0.001
T3	4.998 (3.872, 6.451)	< 0.001	8.831 (6.256, 12.466)	< 0.001	8.421 (5.701, 12.440)	< 0.001

In comparison to non-END.

Early neurological deterioration: END; Triglyceride-glucose index: TyGI; Confidence interval: CI; Odds ratios: OR.

T1/T2/T3: TyGI < 8.804, 8.804 ≤ TyGI < 9.486, and ≥ 9.486, respectively.

^a^Unadjusted-Model 1.

^b^Sex and age-adjusted Model 2.

^c^Sex, age, BMI, smoking, CRP, HCY, DM, HTN, HDL, and NIHSS-adjusted Model 3.

### TyG-BMI and END association

Subsequently, we discovered that TyG-BMI exhibited independence as an END development risk factor (*P* < 0.05) (Table 4). In models 4 and 5, the risk of END was 6.060- (95% CI: 4.668–7.866, *P* < 0.001) and 5.994-fold (95% CI: 4.603–7.805; *P* < 0.001) increased, respectively, in B3 group in comparison to B1 group when TyG-BMI was categorized into B1/2/3 groups. The B3 group was 5.475 times larger than the B1 group (95% CI: 4.157–7.163, *P* < 0.001) in the sex, age, DM, HTN, CRP, HCY, NIHSS, smoking, and HDL-adjusted model 6.

**Table 4:** Triglyceride glucose-body mass index (TyG-BMI) and early neurological deterioration (END) association.

Variables	END					
	OR (95% CI)^a^	*P*-value	OR (95% CI)^b^	*P*-value	OR (95% CI)^c^	*P*-value
TyG-BMI index	1.019 (1.017, 1.022)	< 0.001	1.019 (1.017, 1.022)	< 0.001	1.019 (1.016, 1.021)	< 0.001
B1	Reference					
B2	1.638 (1.259, 2.130)		1.651 (1.268, 2.148)	< 0.001	1.669 (1.274, 2.188)	< 0.001
B3	6.060 (4.668, 7.866)		5.994 (4.603, 7.805)	< 0.001	5.475 (4.157, 7.163)	< 0.001

In comparison to non-END.

Confidence interval: CI; odds ratios: OR.

B1/B2/B3: TyGI < 219.881, 219.881 ≤ TyGI < 262.937, and ≥ 262.937, respectively.

^a^Unadjusted-Model 4.

^b^Age and sex-adjusted Model 5.

^c^Sex, age, DM, HTN, smoking, NIHSS, HDL, CRP, HCY-adjusted Model 6.

### METS-IR and END association

Table 5 shows that METS-IR was significantly related to heightened END risk and represented an independent END risk factor (*P* < 0.05). Using group M1 as the reference, in models 7, 8, and 9, END risk in group M3 was 6.090- (95% CI: 4.684–7.919; *P* < 0.001), 6.015- (95% CI: 4.621–7.830; *P* < 0.001), alongside 6.207-fold (95% CI: 4.728–8.149; *P* < 0.001), respectively.

**Table 5:** Insulin resistance metabolic score (METS-IR) and early neurological deterioration (END) association.

Variables	END					
	OR (95% CI)^a^	*P*-value	OR (95% CI)^b^	*P*-value	OR (95% CI)^c^	*P*-value
METS-IR	1.100 (1.085, 1.115)	< 0.001	1.099 (1.085, 1.114)	< 0.001	1.101 (1.086, 1.117)	< 0.001
M1	Reference					
M2	1.919 (1.474, 2.497)	< 0.001	1.924 (1.477, 2.505)	< 0.001	2.087 (1.590, 2.738)	< 0.001
M3	6.090 (4.684, 7.919)	< 0.001	6.015 (4.621, 7.830)	< 0.001	6.207 (4.728, 8.149)	< 0.001

In comparison to non-END.

Confidence interval: CI; Odds ratios: OR.

M1/M2/M3: METS-IR < 35.021, 35.021 ≤ METS-IR < 42.704, and ≥ 42.704, respectively.

^a^Unadjusted-Model 7.

^b^Age and sex-adjusted Model 8.

^c^Sex, age, HTN, DM, smoking, NIHSS, CRP, HCY-adjusted Model 9.

### TG/HDL and END association

Herein, the TG/HDL could also be an independent END risk factor, both as continuous and categorical variables (Table 6). In models 10, 11, and 12, END risk was significantly elevated in group G3 in comparison to groups G1/G2 and was statistically significant in all cases.

**Table 6:** Triglyceride to high-density lipoprotein ratio (TG/HDL) and early neurological deterioration (END) association.

Variables	END					
	OR (95% CI)^a^	*P*-value	OR (95% CI)^b^	*P*-value	OR (95% CI)^c^	*P*-value
TG/HDL	1.181 (1.141, 1.223)	< 0.001	1.201 (1.155, 1.249)	< 0.001	1.153 (1.105, 1.203)	< 0.001
G1	Reference					
G2	1.368 (1.059, 1.767)	< 0.001	1.588 (1.208, 2.087)	< 0.001	2.338 (1.713, 3.191)	< 0.001
G3	4.283 (3.328, 5.511)	< 0.001	5.403 (4.012, 7.275)	< 0.001	6.175 (4.344, 8.777)	< 0.001

Compared with non-END.

Confidence interval: CI; Odds ratios: OR.

G1/G2/G3: TG/HDL < 3.169, 3.169 ≤ TG/HDL < 5.104, and ≥ 5.104, respectively.

^a^Unadjusted-Model 10.

^b^Age and sex-adjusted Model 11.

^c^Sex, age, HTN, BMI, DM, smoking, NIHSS, CRP, HCY-adjusted Model 12.

### The four indicator performance in END risk prediction

**Figure 2:**
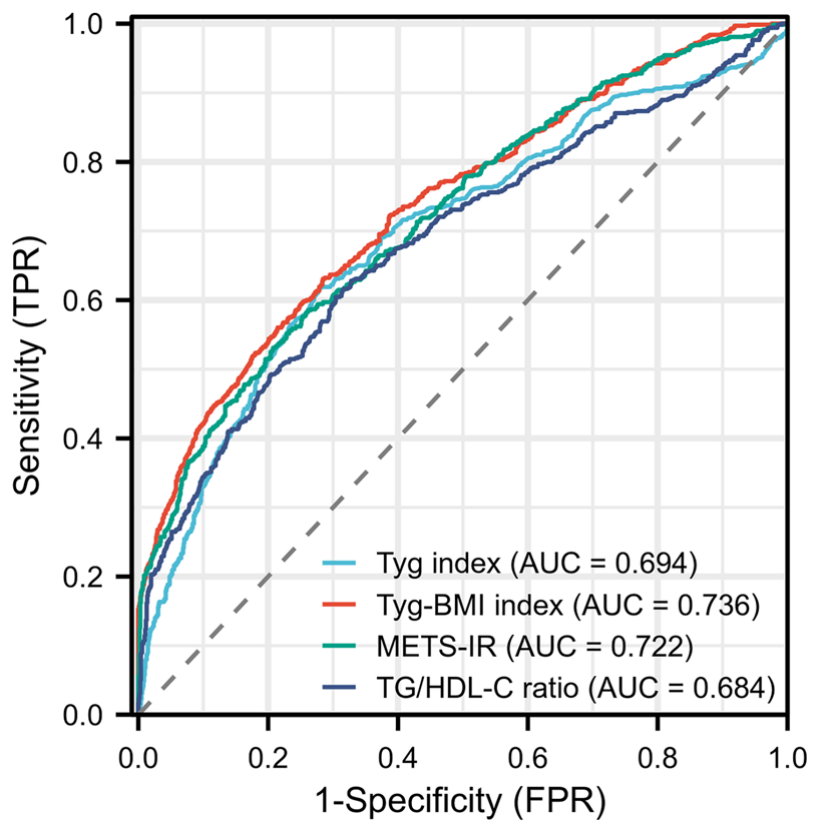
Receiver operating characteristic curve (ROC) curve assessing triglyceride-glucose index (TyGI), insulin resistance metabolic score (METS-IR), triglyceride glucose-body mass index (TyG-BMI), and TG/HDL predictive values for early neurological deterioration (END).

As displayed in Fig. 2, we used the AUC of the ROC curve for evaluating each metric predictive performance. Table 7 summarizes that TyGI and TyG-BMIs’ AUCs were, respectively, 0.694 (95% CI: 0.668–0.721; *P* < 0.001) and 0.736 (95% CI: 0.712–0.761;* P* < 0.001). However, TG/HDL and METS-IR’s AUCs were, respectively, 0.684 (95% CI: 0.658–0.711; *P* < 0.001) and 0.722 (95% CI: 0.697–0.747; *P* < 0.001). Comparing the AUC using MedCalc showed that the AUC of TyG-BMI was slightly higher than the other measures (all *P* < 0.05), suggesting better predictive performance (Fig. 2).

**Table 7:** Performance of four metrics for early neurological deterioration prediction.

Variable	AUC	95% CI	Sensitivity (%)	Specificity (%)	Accuracy (%)
Triglyceride-glucose index	0.694	0.668–0.721	0.618	0.720	0.679
Triglyceride glucose-body mass index	0.736	0.712–0.761	0.631	0.716	0.682
Insulin resistance metabolic score	0.722	0.697–0.747	0.576	0.747	0.679
Triglyceride to high-density lipoprotein ratio	0.684	0.658–0.711	0.604	0.696	0.659

The area under the curve: AUC; confidence interval: CI.

## Discussion

To our knowledge, our study is the first to examine the impact of four promising IR metrics on END, based on a case with a relatively large sample size. Additionally, we are the initial effort for comparing TG/HDL, TyGI, TyG-BMI, along METS-IR as END risk predictors in middle-aged and older AIS patients.

END—common in the acute stroke phase—affects around 10–40% of patients^24^ and possesses a direct association with poor long-term outcomes post-stroke^25^. Previous studies have associated body measurements (e.g., BMI and waist circumference) and IR-related measurements with the incidence and prevalence of stroke, which are mutually reinforcing^26,27^. IR induces several metabolic disorders that promote atherosclerotic plaque rupture, constituting an important risk factor for stroke onset and progression^28^. The normoglycemic clamp test—a gold standard for IR—is slow, complex, and relatively expensive, limiting its use in clinical practice^29^. New, simple, and effective alternative indicators are therefore urgently needed. Composite indices, including METS-IR, TyGI, and TG/HDL (derived from routine hematology studies), have been elucidated to be effective proxies for IR levels with a strong correlation with stroke development.

Our study found that the previously mentioned four indicators exhibited the most significantly elevated levels in the END group. When grouped using tertiles, these four indicators represented independent risk factors for END occurrence, whether or not they were adjusted for confounding factors. Furthermore, as the tertile of each measure rose, the risk of END gradually increased. In this way, our study has identified new predictors that are easy to monitor and can predict END. By monitoring changes in the above indicators, it is hoped that early identification and timely intervention in high-risk groups will reduce the incidence of END.

TyG-BMI is a novel infrared marker combined with the anthropometric measure BMI^30^. A large prospective study represented a correlation between TyG-BMI and an elevated stroke risk^26^. Furthermore, a national prospective cohort study manifested that alterations in TyG-BMI were independently linked to the likelihood of having a stroke in elderly and middle-aged individuals. Monitoring long-term fluctuations in TyG-BMI could prove valuable in identifying individuals with a higher stroke risk^20^. However, no research has examined TyG-BMI and END risk correlation; accordingly, we focused on examining this association. Our findings revealed that these two indicators are independently correlated, even following the adjustment for potential confounding variables. Notably, a rise in TyG-BMI is related to an escalation in END risk. Therefore, TyG-BMI can serve as a valuable indicator for promptly identifying END occurrences in elderly and middle-aged AIS patients.

Furthermore, the TyGI constitutes a surrogate IR marker, helping to identify vascular disease early^31,32^, which represented an independent predictor of stroke progression in a study conducted in a large community center in the United States^28^. Besides, our previous research reported that the TyGI was a significant END risk factor with reliable predictive power^33^. Consistently, TyGI has been demonstrated as an independent END development risk factor, with the two highest quartiles having a higher risk than the lowest quartile.

The TG/HDL—an unconventional lipid parameter—has a strong association with cerebrovascular disease, offering innovative insights into the balance between atherogenic and antiatherogenic lipids^34^. A study found a positive association with adverse outcomes in AIS patients having TG/HDL exceeding 3.515^35^. Although no study has correlated this previously mentioned ratio with END, our study elucidated that this ratio could be an independent END predictive factor with a high predictive value that was similar to TyG-BMI and the TyGI. Accordingly, this predictive ability must be confirmed in further studies.

METS-IR, developed by Bello-Chavolla and colleagues^36^, is a novel IR marker that combines glucose and lipid metabolism with the state of nutrition. METS-IR has a close relation to several risk factors for stroke, including DM, obesity, HTN, and atherosclerosis^37–39^. An increased METS-IR can help identify people at a higher risk of experiencing a stroke^40^. In addition, it was discovered that METS-IR exhibits a correlation with a higher poor prognosis post-intravenous thrombolysis^41^. Regrettably, METS-IR and END correlation has yet to be studied. Nonetheless, we indicate that METS-IR can predict END occurrence and may be considered an independent END risk factor. Furthermore, AUC was higher in comparison to that of the TyGI and TG/HDL, and future prospective studies must further investigate the application of these measures.

Our study is limited to the following: First, as a single-center, large-sample, retrospective study from Northeast China, its generalizability to the population is uncertain. Second, this study did not record patients’ medication history and history of atrial fibrillation, which may have affected the results, but we used multivariate LRA to control for confounders in our analyses, which somewhat reduced the effect of these factors. In addition, by analysing only stroke patients on general wards and excluding those on stroke units, there was some bias in the selection of the population. Therefore, we will conduct prospective, multi-center studies that include more variables to evaluate the relation between these indicators and END more closely.

## Conclusion

In summary, METS-IR, TyGI, TyG-BMI, as well as TG/HDL were significantly related to END occurrence and were important markers for predicting END. By monitoring these indicators, healthcare decisions and risk management for AIS patients can be improved in advance.

## Data Availability

Original data can be obtained by contacting the corresponding author if reasonably requested.

## Abbreviations

IR: Insulin resistance
AIS: Acute ischemic stroke
TyG-BMI: Triglyceride-blood glucose body mass index
END: Early neurological deterioration
TyG: Triglyceride and glucose
TG/HDL-C: Triglyceride to high-density lipoprotein cholesterol
METS-IR: Metabolic score for insulin resistance
ROC: Receiver operating characteristic
AUC: Area under the curve
FBG: Fasting blood glucose
TG: Triglyceride
BMI: Body mass index
TC: Total cholesterol
HDL-C: High-density lipoprotein cholesterol
LDL-C: Low-density lipoprotein cholesterol
CRP: C-reactive protein
HCY: Homocysteine
NIHSS: National Institutes of Health Stroke Scale
OR: Odds ratios
CI: Confidence intervals
DM: Diabetes mellitus
